# Bone marrow edema of the knee: a narrative review

**DOI:** 10.1007/s00402-024-05332-3

**Published:** 2024-04-20

**Authors:** Eleonora Villari, Vitoantonio Digennaro, Alessandro Panciera, Riccardo Ferri, Lorenzo Benvenuti, Faldini Cesare

**Affiliations:** 1st Orthopaedic and Traumatologic Clinic, IRCCS Istituto Ortopedico Rizzoli, Via G.B. Pupilli 1, Bologna, 40136 Italy

**Keywords:** Bone marrow edema, Knee review, Core decompression – subchondroplasty, Total knee arthroplasty – unicompartmental knee arthroplasty

## Abstract

Bone marrow edema (BME) is a frequent MRI finding in patients with knee pain. According to the etiology, BME of the knee can be classified into three main categories: ischemic, mechanic, and reactive. The diagnosis may be difficult, because of the specificity of symptoms and the poor radiographic findings. MRI is the gold standard, showing an area of altered signal of the bone with an high signal intensity on fat-suppressed, T2 weighted images, usually in combination with an intermediate or low signal intensity on T1 weighted images. Bone marrow edema tends to be self-limiting and, in most cases, resolves without any consequences in a varying amount of time. However, since it may evolve to complete joint destruction, early diagnosis and correct treatment are crucial to prevent the articular degeneration. Conservative therapy is the first step, with no weight-bearing for 3 to 6 weeks on the affected side, in combination with the administration of anti-inflammatory drugs or painkillers to manage symptoms. In non-responding forms and more advanced stages, minimally invasive preservative surgery can provide significant results, with subchondroplasty and core decompression being the two main procedures available. Knee arthroplasty, both total (TKA) or unicompartmental (UKA), is the only effective option when the degradation of cartilage is diffuse and in patients with subchondral bone collapse.

**Supplementary Information:**

The online version contains supplementary material available at 10.1007/s00402-024-05332-3.

## Introduction

Bone marrow edema (BME) is frequently found in individuals with knee pain [1] with a prevalence of 17% in young adults [2]. It can be identified on the MRI as an area of altered signal of the bone with a high signal intensity on fat-suppressed, T2 weighted images, usually in combination with an intermediate or low signal intensity on T1 weighted images [3]. Typically, BME is a non-specific MRI finding that can be the result of a variety of different mechanisms and can be found in several clinical diseases with different histological features [4]. The main symptom of BME in the knee joint is pain, due to the increasing intraosseous pressure, which is exacerbated by weight loading and during the night [3]. Bone marrow edema lesions (BML) tend to be self-limiting and, in most cases, resolve without any consequence in a varying amount of time [1]. On the other hand, BML in OA patients has been associated with the development of cartilage damage, a worsening of joint derangement, and, in the worst scenarios, the need for joint replacement [5]. This narrative review aims to examine etiology, diagnosis, and current treatment options for BME with a focus on knee pathology.

### Etiology

According to the etiology, BME of the knee can be classified into three categories (Table 1).

**Table 1:** Causes of BME of the knee

1. Ischemic	Osteonecrosis • Spontaneous (SPONK) • Secondary (SONK) • Post-arthroscopic (PAONK) Bone marrow edema syndrome (BMES) Osteochondritis dissecans (OCD) Complex regional pain syndrome (CRPS)
2. Mechanical	Bone contusion (bone bruise) Stress-related BME Stress fractures • Fatigue fractures • Insufficiency fractures
3. Reactive	Gonarthritis Osteoarthritis Postoperative BME Tumor-related BME

### Ischemic BME

Ischemic bone marrow edema results from a reduction of perfusion [6].

#### Osteonecrosis

In osteonecrosis of the knee, it is possible to distinguish between three different forms: spontaneous (SPONK), secondary (SONK), and post-arthroscopic (PAONK) [7]. *Spontaneous osteonecrosis (SPONK)* is the consequence of subchondral insufficiency fracture [7]. Usually regards patients older than 55 years, predominantly females, without particular risk factors [7]. Clinically the onset is characterized by acute knee pain in the antero-medial compartment, exacerbated by movement and worst at night, and typically coexists with meniscal tears [7]. The initial stage is reversible, however, when cartilage degeneration progresses, osteoarthritis may develop [8]. Some authors believe that SPONK represents a BMES secondary to a subchondral insufficiency fracture rather than a true osteonecrosis [9]. *Secondary osteonecrosis* (SONK) affects younger patients, from 20 to 55 years, with typical risk factors such as alcohol abuse, obesity, and corticosteroid use or specific clinical conditions like sickle cell disease, myeloproliferative disorders, and Gaucher’s disease [10]. *Post-arthroscopic osteonecrosis* (PAONK) is the least common and may occur in older patients following arthroscopic surgery, especially meniscectomy and chondroplasty [7]. It is a rare condition, with an incidence of 0.2–1.5% considering knees underwent arthroscopic surgery [11].

#### Bone marrow edema syndrome (BMES)

BMES is a transient condition with a bone marrow edema pattern, mainly characterized by its self-limited nature which generally affects middle-aged males [12]. It is defined by typical BME features at MRI along with clinical symptoms like pain, decreased joint motion, and increased interstitial fluid without a clear cause and no obvious signs of osteonecrosis, trauma, or infection [13].

#### Osteochondritis dissecans (OCD)

OCD is a focal idiopathic alteration of subchondral bone, that primarily affects adolescent patients [14]. The osteochondral fragment is unstable and it is at risk of detachment with subsequent progression to osteoarthritis [14]. In 80% of cases, the main symptom is pain during weight bearing [15]. Transient quadriceps blocks may cause the knee to buckle in patients with an unstable or loose fragment [15].

#### Complex regional pain syndrome (CRPS)

Also known as algodystrophy or Morbus-Sudek syndrome, is a disorder that might occur after an unidentified trauma or injury [6]. Symptoms are persistent burning pain, trophic disruptions, and sensory changes [6]. In contrast to BMES, approximately one-third of patients with CRPS evolve to chronicity [16].

### Mechanical BME

Mechanical BME is the most frequent since it is mainly related to trauma [6]. In these cases, it is generally referred to as “*bone bruise*” [17]. Both acute trauma and chronic repeated stresses may lead to a breakdown of the marrow trabeculae, resulting in interstitial fluid leakage and hemorrhage to marrow spaces [5].

#### Bone contusion (bone bruise)

**Fig. 1:**
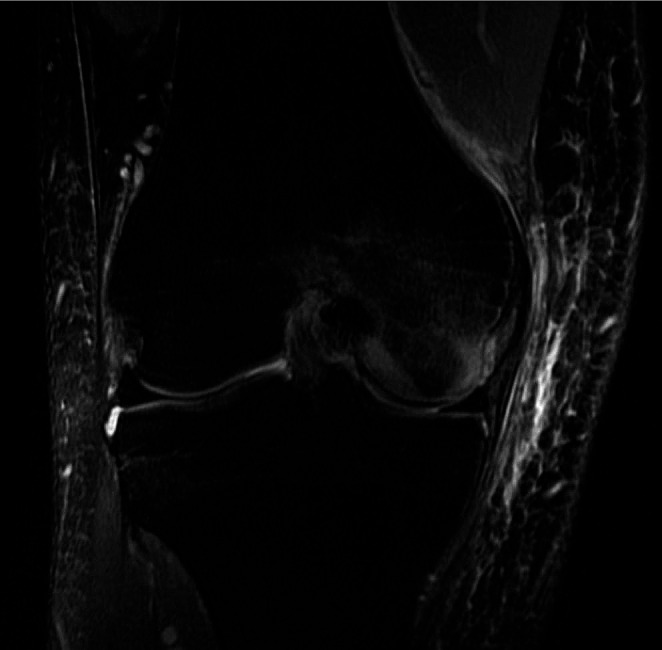
Post-traumatic BME of the medial femoral condyle

It is a trabecular microfracture resulting from an impaction, distraction, or direct trauma [17]. The most frequent patterns that may result in BME are those triggered by acute anterior cruciate ligament (ACL) rupture, transitory lateral patellar dislocation, and varus and valgus injuries [17] (Fig. 1).

#### Stress-related BME

Is due to an overload of subchondral bone, generally related to malalignment or mechanical stress [6]. In those patients, BML can be found in the overloaded compartment, with the medial femoral condyle being the most affected [6].

#### Stress fracture

Stress fractures include fatigue and insufficiency fractures [18]. Repetitive overload of normal bone structures can result in a fatigue fracture, which typically regards runners and other athletes [18]. Instead, insufficiency fractures are the result of normal or traumatic loading on abnormal bone: they are generally spontaneous, without any trauma or overload, and develop in a pathologic bone tissue such as osteoporotic bone in elderly populations [18].

### Reactive BME

Reactive bone edema is a concomitant condition associated with an underlying disease [19]. It is brought on by an inflammatory response to a contiguous process, such as intra-articular infection, osteomyelitis, inflammatory arthritis, tumors, or previous surgeries [19].

#### Gonarthritis

A wide variety of arthritis conditions can be accompanied by bone marrow edema, for example, reactive arthritis, bacterial arthritis, and osteomyelitis [6]. In those cases, patients often present systemic symptoms like fevers or sepsis, limited range of motion of the knee, and difficulty ambulating [19].

#### Osteoarthritis

Bone marrow edema is a common finding in knee osteoarthritis; in the final stages, osteoarthritis tends to show joint effusion, subchondral edema, geodes, and reactive synovitis [20]. Bone marrow lesions in knee osteoarthritis can change in size quickly and are associated with the progression of articular cartilage degradation and pain exacerbation [21]. As demonstrated, patients with osteoarthritis commonly experience a higher level of pain when BME is present [22] and it is also considered a poor predictor of the progression of the disease [23].

#### Postoperative BME

**Fig. 2:**
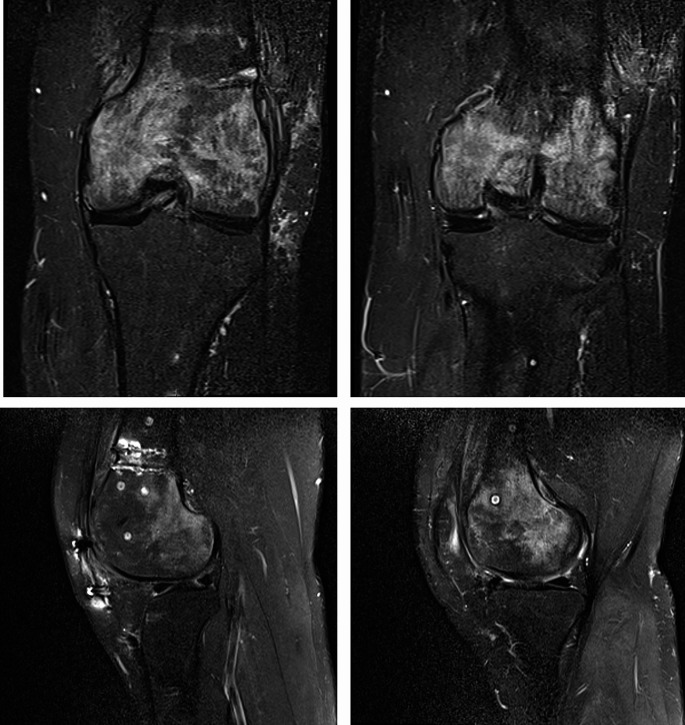
BME at MRI in a 29 year old male after trocheoplasty, femoral varus osteotomy, and anterior tibial tuberosity transposition

Postoperative BME in the knee joint can be observed after drilling, ligament reconstructions, and surgical procedures involving the osteochondral compartment [6]. BMLs are detectable around and above the treated site and can occur up to six to twelve months following surgery [1]. It is manifested as a persisting or recurrent pain [1] (Fig. 2).

#### Tumor-related BME

Also if tumors around the knee are uncommon, both benign and malignant tumors may be followed by a reactive bone edema [6]. The most commonly occurring are osteosarcoma, chondrosarcoma, and Ewing sarcoma which typically affect children [24]. Considering benign lesions, the main painful bone lesion that can mimic BME is osteoid osteoma, occurring in children and young adults [24].

### Diagnosis

#### Clinical features and physical examination

The first symptom in patients with bone marrow edema is knee pain involving the affected side, which is typically acute, disabling, and exacerbated by weight-bearing [8]. Another common sign is ache while tapping the afflicted area. The majority of patients describe the onset of the pain as spontaneous, however, it could be also associated with a minor trauma [8] while its intensity varies too, shifting from being vague and subtle to developing quickly into a severe discomfort that can led to immobilization [13]. A further aspect of the pain is that it frequently persists during the night. However, sometimes BME can also be found in asymptomatic patients [25]. Clinically, the knee might be swollen and patella ballottement is positive [8]. The joint range of motion (ROM) is mostly preserved [26].

### Imaging

The first-level examination is standard radiography in anterior-posterior (AP) and lateral (LL) projections, however, in the early stages of the disease, it is unable to detect structural changes which start to show up about three to six weeks after the symptoms first arises [27]. When visible, BME can be identified as an area of variable focal loss of bone density and blurring of the trabecular structure, with poorly defined cortical borders [5, 28]. The medial femoral condyle is more frequently involved than the lateral, followed by tibia and patella [18]. MRI is the exam of choice for early detection of BME, permitting edema identification as early as 48 h from the onset of symptoms [14]. Fat-saturated sequences, such as PDW FS or STIR, should be included in the sequencing procedure for BME in three different spatial orientations [14]. The pattern of bone marrow edema at MRI is determined by the mechanism of injury: shear forces cause an oblique orientation of the bone bruise concerning the direction of the applied force, impaction trauma results in a diffuse edema, avulsion forces produce a linear bone bruise perpendicular to the axis of stress that may also cause an avulsion fracture if the trauma is sufficiently strong [29]. Recently, Dual-energy CT has proven to be effective for the identification of BME in the knee, both in the acute and chronic settings, and it is particularly useful in patients whose conditions preclude them from being evaluated through MRI [30].

### Differential diagnosis

A variety of conditions has to be considered in the differential diagnosis, such as injuries like contusions and fractures, the development of cysts and erosions, and issues related to the bone marrow like hematopoietic changes and infiltrations [1]. When patients present systemic symptoms and an inflammatory arthritis is suspected, an intra-articular aspiration should be performed to sample local fluid to assess the gram stain, crystal presence, and synovial fluid cell count [19].

### Treatment

**Fig. 3:**
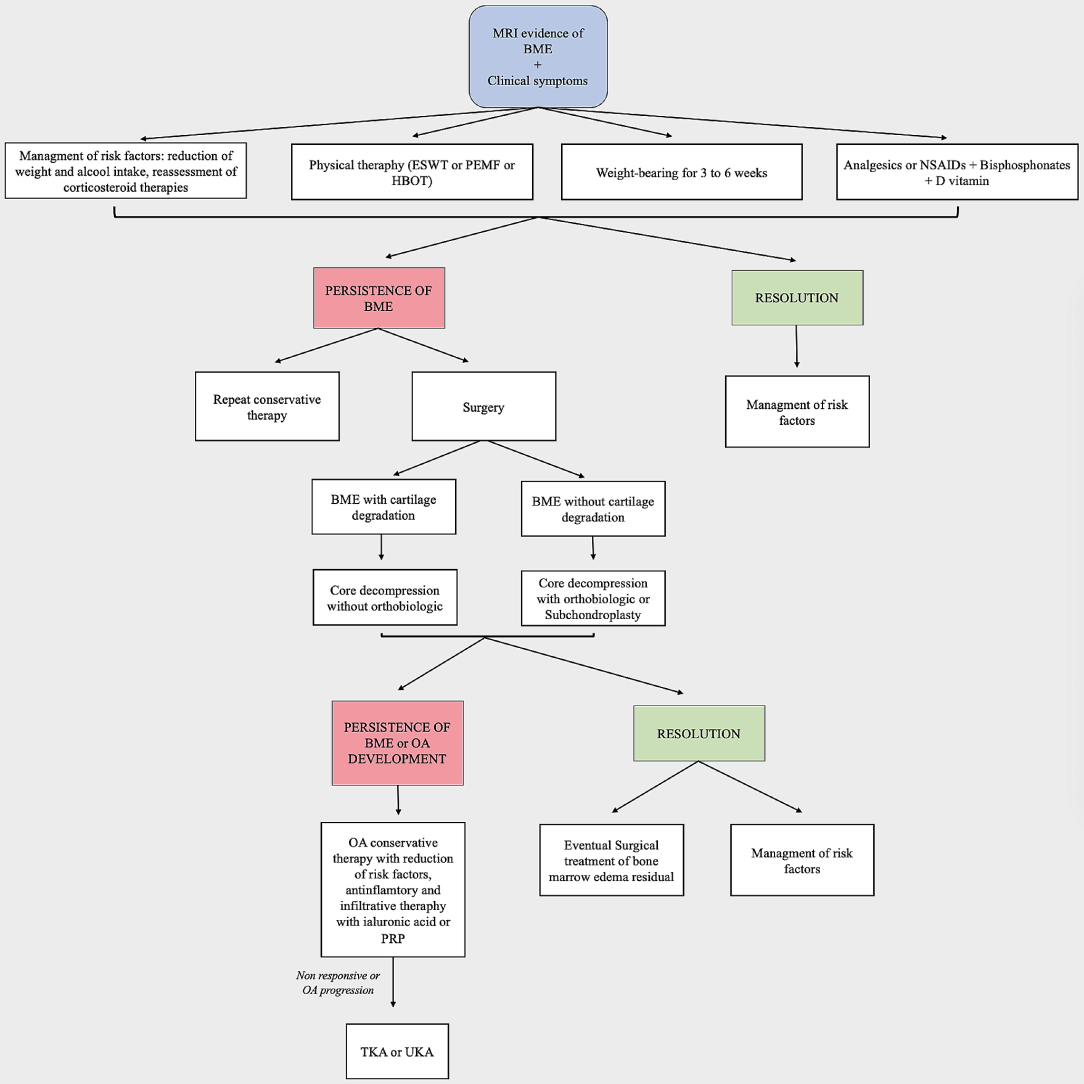
Therapeutic algorithm of BME of the knee. ESWT: extracorporeal shock waves, PEMF: pulsed electromagnetic field, HBOT: hyperbaric oxygen therapy, NSAIDs: Nonsteroidal anti-inflammatory drug, OA: osteoarthritis, PRP: plated-rich plasma, TKA: total knee arthroplasty, UKA: unicompartmental knee arthroplasty

Bone marrow edema of the knee is typically self-limiting and primarily requires pain management [1]. It is generally recommended to avoid weight-bearing for 3 to 6 weeks on the affected side, in combination with the administration of anti-inflammatory drugs or painkillers to manage symptoms [6]. Pharmacological and physical therapy are also included in conservative management [6]. Surgery may be necessary if conservative treatment is unsuccessful [31] (Fig. 3).

### Pharmacological treatment

Conservative treatment begins with pain management [6]. Analgesic and NSAIDs are widely used [6]. In particular, acetylsalicylic acid (ASA) can be particularly useful in the early stages of disease if administrated daily, based on its anticoagulant attributes, as well as its ability to protect and positively modulate the vascular endothelium [32].

Patients with BME can benefit from the administration of bisphosphonates and vitamin D supplementation [33]. They work by decreasing osteoclast-mediated bone turnover, helping to prevent subchondral bone collapse [33]. Among the bisphosphonates, Ibandronate (intravenous), Alendronate (oral), Pamidronate (intravenous), Neridronate (intravenous), and Clodronate (intramuscular) have demonstrated effectiveness [33]. Ibandronate showed a significant improvement in pain management as compared to standard therapy of analgesics and reduced weight bearing, if administered intravenously in one to three doses of 3 mg, depending on the severity of pain and treatment response [34]. It is necessary to monitor vitamin D and calcium levels and administer supplementation as needed to lower the risk of hypocalcemia [35]. Alendronate, when administered orally at a dosage of 35 mg twice a week for 16 weeks in association with single a dose of 5 mg of intravenous zoledronic acid, showed early pain relief and reduction of the bone edema, while being well-tolerated [36]. Also Neridronate, intravenously administered in 4 doses of 100 mg, has proven to be effective [37].

Prostacyclins, through arterial and venous vasodilatation, improve microcirculation consequently it can be used not only to reduce BME but also to improve accompanying symptoms [38]. Particularly, the analogous Iloprost has shown to be helpful in the conservative treatment of ischemic and post-traumatic BME [38]. Iloprost shall be administered in intravenous infusion of 20 µg/ml in 500 ml of sodium chloride solution, given over a period of 6 h for 5 consecutive days [39].

### Physical therapy

*Extracorporeal shockwave therapy (ESWT)* can be used in bone marrow edema lesions to improve pain and function and to accelerate the normalization of the MRI signal [40]. It works by stimulating angiogenesis and osteogenesis through osteoblasts, periosteal cells, and vascular endothelial growth factor. ESWT demonstrated a reduction in pain and an increase in patient reported function. especially at 3 and 6 months follow-up, if provided in two to three sessions at weekly intervals or every three weeks [40].

*Hyperbaric oxygen (HBO)* is also a safe and non-invasive option [41]. The procedure relies on the exposure to high levels of oxygen which raises hemoglobin saturation and improves oxygen transport to the tissues [42]. HBO showed promising results in pain management and return to movement when administered once a day, 5 days a week for 1 month in association with acetylsalicylic acid and bisphosphonates [41].

*Pulsed electromagnetic field (PEMF)* may accelerate the healing process [43]. It increases bone mass by promoting osteoblast proliferation and differentiation and has proven to be effective in decreasing knee pain and the necrosis region during the first six months, maintaining 86% of knees from prosthetic surgery at 24-month follow-up [43]. Pulsed electromagnetic field stimulation is usually prescribed 8 h a day for 30 days [44].

### Infiltrative therapy

*Platelet-rich plasma (PRP)* has been used via intra-articular injections in individuals suffering from both osteoarthritis and BME of the knee [45]. A study showed a significant reduction in subchondral edema and the level of inflammatory biomarkers in synovial fluid in symptomatic patients [45].

*Mesenchymal stem cells* (MSCs), obtained from adult bone marrow (BM-MSC) or adipose tissue (AD-MSC), has been recently included in possible treatment strategies via intra-articular injection [46]. The benefits depend on the MSCs ability to regulate a variety of trophic factors that help prevent oxidative damage, apoptosis, and inflammatory pathways [46].

### Surgical treatment of bone marrow edema

When conservative management is unsuccessful, surgery may be required to alleviate discomfort and enhance function [31]. Subchondroplasty and core decompression are the two main procedures available [31].

**Fig. 4:**
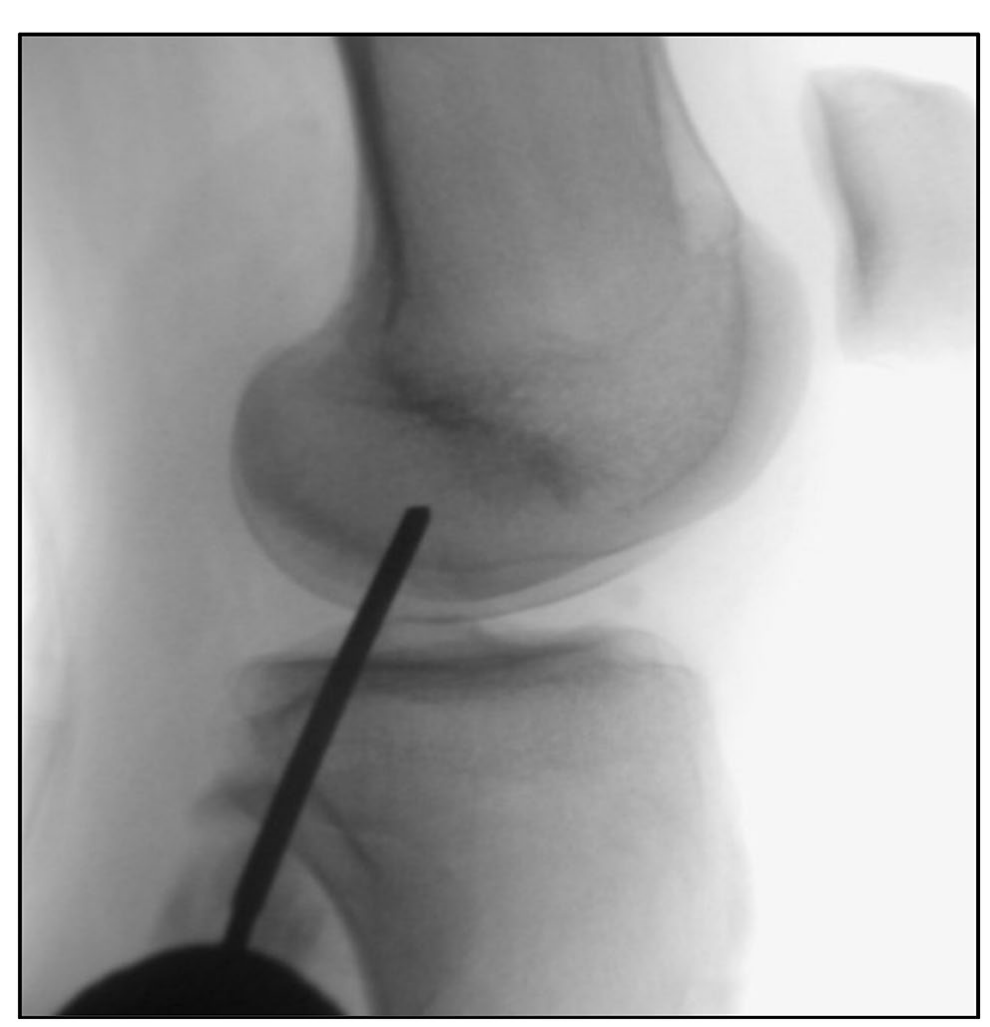
Core Decompression

*Core decompression (CD)* is performed with a percutaneous distal femoral incision on the affected side through a drilling of the area with a bone marrow lesion [47]. The procedure is reserved for early-stage osteonecrosis BME without joint space destruction and is particularly effective in secondary osteonecrosis [48]. It is based on reducing the intraosseous hypertension, caused by the necrosis process and the influx of inflammatory cells into the damaged areas, under 30mmHg [48], which can result in rapid pain relief [49]. Usually, the bone edema is undetectable at MRI three months after surgery [31]. However the residual cavity creates a weaker area at risk of collapsing and CD, when performed alone, reported a failure rate of up to 77% [50, 51]. For this reason, core decompression has been combined with intraosseous injection of bone graft materials which are stuffed into the cavity to improve the effectiveness [51]. Autologous conditioned plasma (ACP) [47], bone grafting and bone marrow aspirate concentrate [52] and β-tricalcium phosphate [53] are the most used (Fig. 4).

**Fig. 5:**
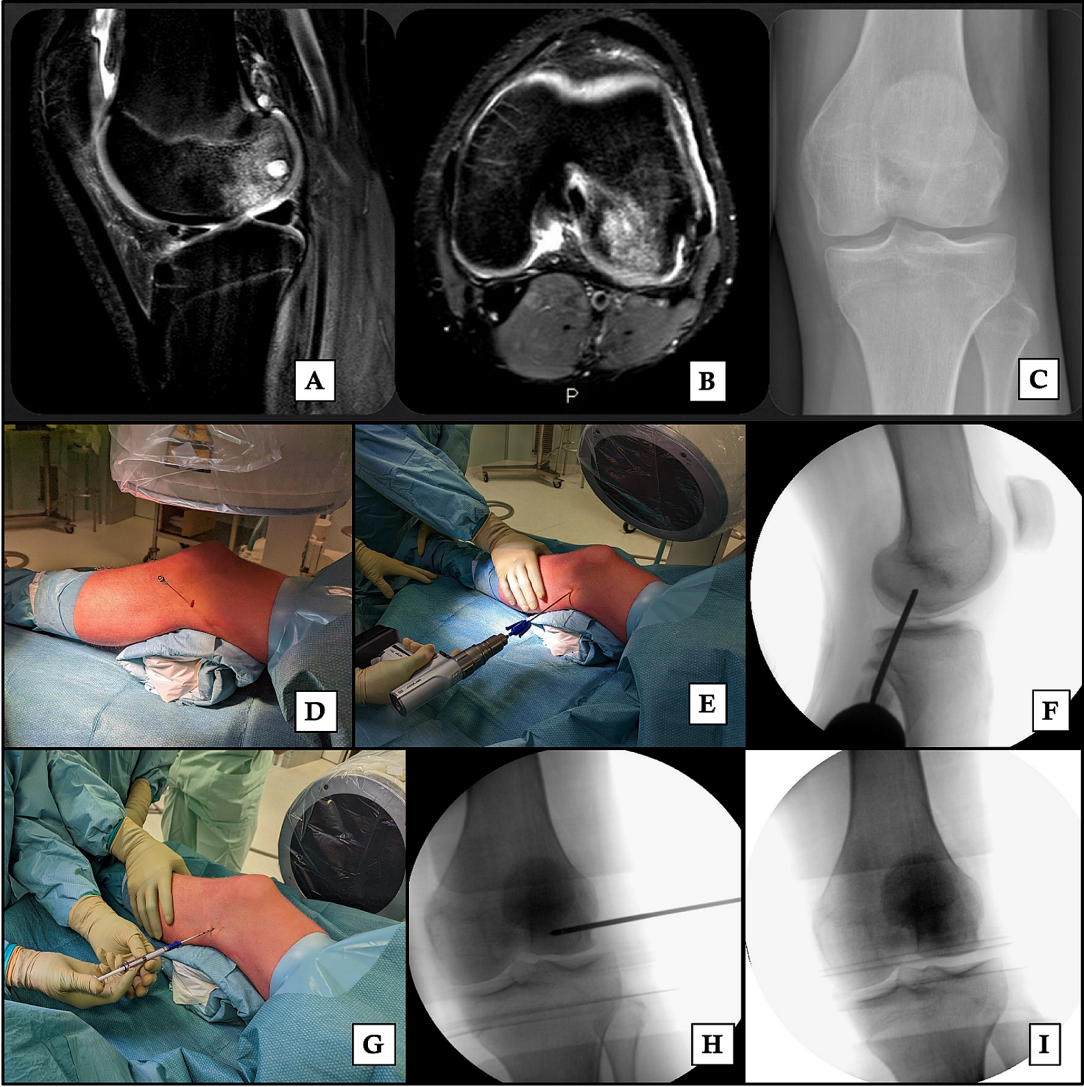
26 years male with a BME of the lateral femoral condyle at MRI (**A**, **B**) and X-Ray (**C**) undergoing subchondroplasty. The guide pin is placed under fluoroscopic guidance (**D**), the fenestrated cannula is inserted inside the lesion (**E**) and then controlled under fluoroscopic guidance (**F**). The syringe, pre-filled with calcium phosphate, is placed (**G**) and controlled under fluoroscopic guidance (**H**). The calcium phosphate is then injected (**I**)

*Subchondroplasty (SCP)* is a variation of the core decompression, developed in 2007, where the defect is stuffed with an orthobiologic: calcium phosphate (CaP) [54]. It consists of an injection of calcium phosphate (between 5 and 16 mL) through a fenestrated cannula inserted over a guide pin, previously placed by fluoroscopic guidance during the procedure or tibial navigation guide [54]. The injection is continued until the fluoroscopic image shows a darkened blush [55] (Fig. 5). After that a standard arthroscopy is generally performed to improve the accuracy of the targeted injection site and for the treatment of any concurrent lesion that is discovered, such as debridement chondroplasty, meniscal repair, or meniscectomies, synovectomy, cyst drainage, and removal of loose bodies [54]. SCP is used to treat persistent, non-healing BML [56]. The two primary goals are enhancing the potential for subchondral bone remodeling and restoring the structural integrity of injured subchondral bone [55, 56].

Another variation of core decompression is *percutaneous drilling*, a technique consisting of performing multiple small percutaneous 3 mm drillings on the BML [57]. This technique, as well as core decompression, has proven to be effective in delaying more invasive surgery in patients suffering from secondary osteonecrosis of the knee [58].

### Surgical treatment of BME residual

Bone marrow edema may resolve without sequelae, however, in some cases, it can result in cartilage defects that need to be treated to restore articular surface integrity and slow the progression to osteoarthritis [23]. In this setting, regenerative medicine plays a key role [59].

In conditions like OCD, in addition to drilling, arthroscopic fixation of the fragment may be necessary [15]. Moreover, if the fragment is too severely damaged to be repositioned or is detached and not found the defect can be treated with micro-fractures, mosaic osteochondral transplantation, or autologous chondrocyte transplantation [15].

Also, regenerative medicine is gaining importance, especially in young adults. It includes procedures such as *osteochondral autograft Transplant* (OAT), *fresh osteochondral allograft* (FOCA), *autologous chondrocytes implantation* (ACI), and *cell-free scaffolds*, depending on the size of the lesion [60]. Small lesions can be treated with OAT, while larger lesions (> 2 cm^2^) may require a cell-based or a cell-free osteochondral scaffold [60]. The last two, however, allow reconstruction with suboptimal tissue quality [60]. Considering this, fresh allografts may currently represent the best alternative because it enables the acquisition of an articular surface repair with viable physiologic osteochondral tissue [60].

Realignment could also be taken into consideration for young patients with stress-related BME and mechanical axis malalignment [61]. In fact, BMLs are more frequently found in the medial compartment, especially in patients with varus malalignment and early osteoarthritis, suggesting a malalignment-related overload of the subchondral bone [61]. Those patients can benefit from a *high tibial osteotomy* and early lateral closing wedge osteotomy has to be considered in individuals with varus malalignment and bone marrow edema, even with mild medial osteoarthritis, due to the prognostic significance of bone marrow abnormalities in the medial compartment of the knee [62].

**Fig. 6:**
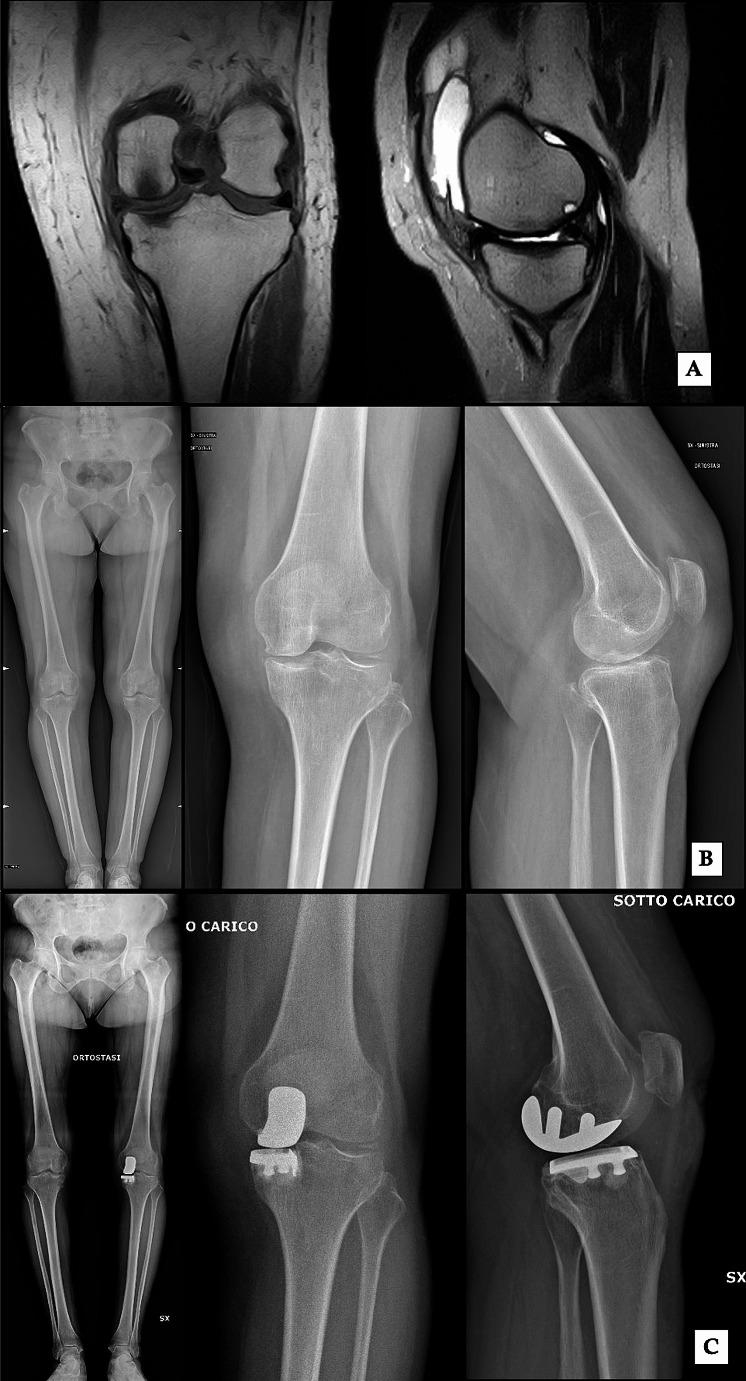
BME of the medial femoral condyle and subchondral cyst in a 70 year female patient at T2 MRI sequences (**A**) and X-Ray before (**B**) and after (**C**) medial unicompartmental knee arthroplasty

*Arthroplasty* may become necessary when the degradation of cartilage is diffuse and in patients with subchondral bone collapse, both total (TKA) or unicompartmental (UKA), based on the extent and the type of lesion [63]. The aim of the procedure is to restore the joint anatomy to provide a good symptomatic and functional outcome [64]. UKA may represent a solution when only one compartment is involved [65] (Fig. 6). However results are conflicting and indication is still debated: some authors reported good outcomes using UKA, with a 95% survivorship at 15-year follow up [66], and claimed that medial tibial BMLs should not be considered a contraindication for medial UKA [63, 64], showing long-term results of UKA for osteonecrosis comparable to UKA for osteoarthritis [67, 68]. On the other hand, others reported higher levels of pain and less satisfaction in patients affected by BME and undergoing UKA compared to TKA [69]. TKA might be the only effective option for patients who have extensive lesions affecting multiple compartments [63]. Better TKA implant fixation in necrotic knee compartments and the absence of secondary arthritic and potential osteonecrotic transformation of further knee compartments appear to be the main advantages of total knee arthroplasty [70].

## Conclusion

Bone marrow edema is a frequent find in painful knee, which might be related or not to underlying diseases. Its non-specific clinical manifestations and the lack of early radiological evidence lead to frequent misdiagnosis. Currently, there is no standard protocol for treating bone marrow edema. However, since it affects young people and may evolve to complete joint destruction, early diagnosis, and correct treatment are crucial to prevent articular degeneration. Conservative therapy is the first step, but in non-responding forms and in more advanced stages, minimally invasive preservative or surgery can provide significant results in symptom relief and function recovery of the patient. Knee arthroplasty, both total (TKA) or unicompartmental (UKA), is the only effective option when the degradation of cartilage is diffuse and in patients with subchondral bone collapse.

### Electronic supplementary material

Below is the link to the electronic supplementary material.

Supplementary Material 1
