# Correction to: Bone marrow edema of the knee: a narrative review

**DOI:** 10.1007/s00402-024-05387-2

**Published:** 2024-06-21

**Authors:** Eleonora Villari, Vitantonio Digennaro, Alessandro Panciera, Riccardo Ferri, Lorenzo Benvenuti, Faldini Cesare

**Affiliations:** 1st Orthopaedic and Traumatologic Clinic, IRCCS Istituto Ortopedico Rizzoli, Via G.B. Pupilli 1, Bologna, 40136 Italy

**Correction to: Archives of Orthopaedic and Trauma Surgery (2024) 144:2305?2316**

10.1007/s00402-024-05332-3

In the original version of this article, the author’s name Vitantonio Digennaro was incorrectly written as Vitoantonio Digennaro.

