# Supplementary material for: Bone marrow edema of the knee: a narrative review
Source: Arch Orthop Trauma Surg. 2024 Apr 20;144(5):2305–16. doi: 10.1007/s00402-024-05332-3 (PMC11093815; doi:10.1007/s00402-024-05332-3)
Supplement: Supplementary file 1 — Supplementary Material 1 [file 402_2024_5332_MOESM1_ESM.docx]

**Statements and Declarations**

- **Funding:** The authors declare that no funds, grants, or other support were received during the preparation of this manuscript
- **Competing Interests:** The authors have no relevant financial or non-financial interests to disclose
- **Author Contributions:** All authors contributed to the study conception and design. All authors contributed to the study conception and design. Material preparation, data collection and analysis were performed by Eleonora Villari, Vitantonio Digennaro, Alessandro Panciera, Riccardo Ferri, Lorenzo Benvenuti and Cesare Faldini.
- **Ethics approval:** no ethical approval is required

The authors have no competing interests to declare that are relevant to the content of this article
